# Evaluation of Factors Influencing Fluoride Release from Dental Nanocomposite Materials: A Systematic Review

**DOI:** 10.3390/nano15090651

**Published:** 2025-04-25

**Authors:** Alicja Morawska-Wilk, Julia Kensy, Sylwia Kiryk, Agnieszka Kotela, Jan Kiryk, Mateusz Michalak, Natalia Grychowska, Magdalena Fast, Jacek Matys, Maciej Dobrzyński

**Affiliations:** 1 Department of Pediatric Dentistry and Preclinical Dentistry, Wroclaw Medical University, Krakowska 26, 50-425 Wroclaw, Poland; alicja.morawska@umw.edu.pl (A.M.-W.); s.roguzinska@gmail.com (S.K.); maciej.dobrzynski@umw.edu.pl (M.D.); 2 Faculty of Dentistry, Wroclaw Medical University, Krakowska 26, 50-425 Wroclaw, Poland; julia.kensy@student.umw.edu.pl; 3 Medical Center of Innovation, Wroclaw Medical University, Krakowska 26, 50-425 Wroclaw, Poland; kotela.agnieszka@gmail.com (A.K.); mateusz.michalak92@gmail.com (M.M.); 4 Dental Surgery Department, Wroclaw Medical University, Krakowska 26, 50-425 Wroclaw, Poland; jan.kiryk@umw.edu.pl; 5 Department of Dental Prosthetics, Wroclaw Medical University, Krakowska 26, 50-425 Wroclaw, Poland; natalia.grychowska@umw.edu.pl; 6 Department of Drug Form Technology, Wroclaw Medical University, Borowska 211 A, 50-556 Wroclaw, Poland; magdalena.fast@umw.edu.pl

**Keywords:** fluoride release, nanocomposites, nanostructured, nanofilled, composite

## Abstract

This systematic review aims to evaluate factors influencing fluoride release from dental nanocomposite materials. A comprehensive database search was conducted in February 2025 using PubMed, Web of Science, and Scopus. The search terms “fluoride release AND nanocomposites” were applied following PRISMA guidelines. Out of 336 initially identified articles, 17 studies met the inclusion criteria and were selected for analysis. Seventeen studies confirmed that fluoride-releasing nanocomposites are effective, with fluoride ion release influenced by material composition, nanofiller type, and storage conditions. Studies showed that acidic environments (pH 4–5.5) significantly enhanced fluoride release, particularly in materials containing nano-CaF_2_ or fluoridated hydroxyapatite, which responded to pH changes. Quantitative comparisons revealed that daily fluoride release values ranged from <0.1 μg/cm^2^/day in commercial composites to greater than 6500 μg/cm^2^/day in BT-based nanocomposites and up to 416,667 μg/cm^2^/day in modified GICs. Additionally, some composites exhibited fluoride recharging capabilities, with materials incorporating pyromellitic glycerol dimethacrylate (PMGDM) and ethoxylated bisphenol A dimethacrylate (EBPADMA) demonstrating prolonged fluoride and calcium ion release after recharge exposure, rather than the highest initial values. Despite releasing lower fluoride levels than conventional GIC and RMGI materials, fluoride-releasing nanocomposites demonstrate significant anti-caries potential and clinical applicability, with some formulations supporting periodontal regeneration and caries prevention around orthodontic brackets. However, the lack of consistency in study protocols—including differences in storage media, sample geometry, and measurement methods—limits direct comparison of outcomes. Therefore, the most critical direction for future research is the development of standardized testing protocols to ensure reliable, comparable results across material groups.

## 1. Introduction

**Figure 1:**
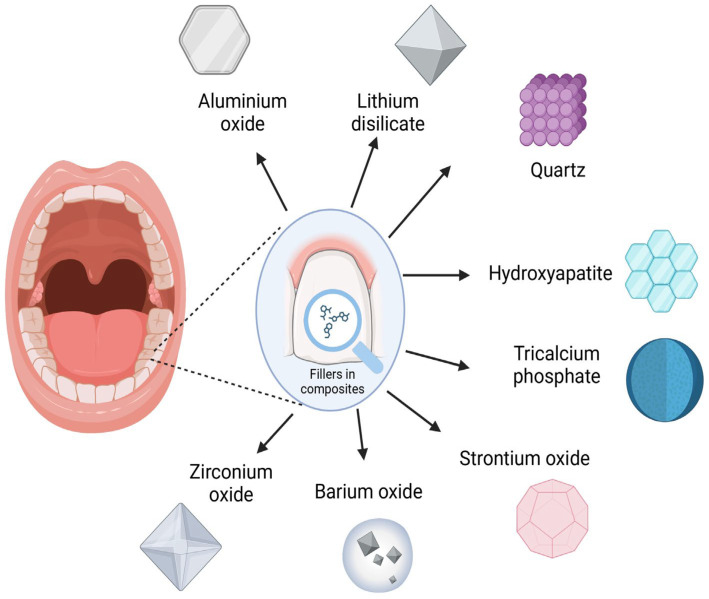
Most commonly used fillers in composite materials (created with BioRender.com).

Composite materials have many applications in modern dentistry, particularly as fillers for lost tooth tissue. An important component of composites is their inorganic component, which acts as a filler and, at the same time, significantly influences the properties of the material [1,2,3]. Filler particles are most commonly quartz, hydroxyapatite, tricalcium phosphate, zirconium oxide, barium oxide, strontium oxide, aluminium oxide, or lithium disilicate [2,3]. In dentistry, they can be referred to as microparticles or nanoparticles, depending on their size [4]. Filler particles in nanocomposites have a specific size, with the smallest particle being in the range of 10–99 nm. Nanocomposites can contain fillers of uniform size or be composed of particles of different sizes (micro + nano); the latter are called nanohybrid composites [2,5]. Nanocomposites are a popular filling material in dentistry because they have better mechanical parameters and aesthetic properties compared to microcomposites. Another important feature is low polymerization shrinkage [6,7,8,9,10]. The addition of nanofillers to the material structure reduces the wear of the composite and the inclusion of compounds such as CHX and CaF_2_ gives it antibacterial properties [11,12,13,14]. In addition, incorporating CaF_2_ into the nanocomposite structure gives the material remineralizing properties and inhibits the demineralization of tooth tissue by releasing Ca-ions and F-ions [15,16] (see Figure 1).

Fluoride release is a highly desirable property in restorative dental materials due to its proven anticariogenic and remineralizing effects. One of the most widely used materials for this purpose is the glass ionomer cement (GIC), composed of an aluminum fluorosilicate glass powder and an aqueous solution of acrylic or polyacrylic acid and maleic acid. The setting reaction is based on a classic acid–base mechanism [17,18]. Conventional GICs and their resin-modified variants (RMGIs) are known for their substantial fluoride ion release, contributing to cariostatic effects. These materials are biocompatible and hydrophilic, and can chemically bond to moist dental tissues. However, they suffer from notable drawbacks such as brittleness, microleakage, low mechanical strength, and suboptimal esthetics [19,20,21,22], limiting their use in long-term restorations. In contrast, fluoride-releasing composite resins offer superior mechanical and esthetic properties, but typically exhibit significantly lower fluoride release compared to GICs, RMGIs, or compomers [23,24]. A promising development in restorative materials is the introduction of nanocomposites, which aim to combine the beneficial fluoride release profile of GICs with the favorable physical characteristics of composite resins. Recent studies suggest that certain nanocomposite formulations can achieve fluoride release levels comparable to those of GICs and RMGIs, while maintaining superior durability and esthetics [19,25].

Fluoride-containing agents are commonly used to prevent and treat dental caries. They can be administered in different ways: endogenously, through the fluoridation of water, milk consumption, tablets, or exogenously, through the use of pastes, gels, foams, and varnishes [26,27,28,29]. The preventive effect of fluoride is to inhibit the demineralization of dental tissues and the remineralization of areas affected by initial carious changes [30,31]. The anticaries mechanism of fluoride ions is based on the inhibition of glycolysis, polysaccharide synthesis, and enolase in bacterial cells, which disrupts their metabolism. The continuous local supply of fluoride compounds within the enamel causes the conversion of hydroxyapatite to fluorapatite, which is more resistant to cariogenic acids [31,32,33,34]. The remineralization mechanism is supported by the attraction of calcium ions to the enamel as a result of fluoride adsorption on the surface of demineralized enamel [32,33,34]. Despite the controversy about the risk of fluorosis and the risk of toxic effects of fluoride on the body [35], the use of fluoride-containing preparations has a significant impact on oral health. Fluoride compounds prevent the development of caries, remineralize tooth tissue in the early stages of caries development, and prevent enamel erosion in people with reflux disease [31,32,33,34,36,37,38].

The integration of fluoride-releasing mechanisms into nanocomposites typically involves incorporating fluoride-containing components such as strontium or sodium fluoride, calcium fluoride, or ytterbium fluoride into either the resin matrix or nanofiller particles [5,15,25,39]. This controlled fluoride release provides localized protection to the tooth structure, addressing a key limitation of traditional composites. The pH of the environment is one of the most important factors affecting the release of fluorine from nanocomposites [24,40,41]. Studies show that acidic conditions significantly increase the release through increased dissolution of fluoride-containing components, which is strategically important because of carious bacteria that produce acids that lower the local pH, triggering the “on-demand” release of fluoride when it is most needed [42,43,44]. Other important factors affecting fluoride release kinetics include the composition and concentration of fluoride-containing components, the hydrophilicity of the resin matrix, and the particle size distribution of the nanofiller [3,45]. Clinical variables such as surface finishing techniques and mechanical stresses have also been shown to modulate fluoride release patterns in laboratory studies [5,11,45].

Understanding the complex factors modulating fluoride release from nanocomposite restorative materials remains a crucial challenge in restorative dentistry. While numerous individual studies have addressed specific aspects of fluoride release, a comprehensive analysis of the many variables affecting this process has been lacking. This systematic review aims to analyze the factors affecting fluoride release from nanocomposite restorative materials, including changes in pH, material composition, nanofiller properties, and clinical conditions. This comprehensive assessment delivers clinicians practical, research-supported strategies to enhance the performance and preventive capabilities of nanocomposite dental materials.

## 2. Materials and Methods

### 2.1. Focused Question

The systematic review followed the PICO framework [46] as follows: In the case of nanocomposite restorative materials (population), what are the factors (investigated condition) influencing the fluoride release (outcome) from these materials compared to other restorative materials (comparison condition).

### 2.2. Protocol

**Figure 2:**
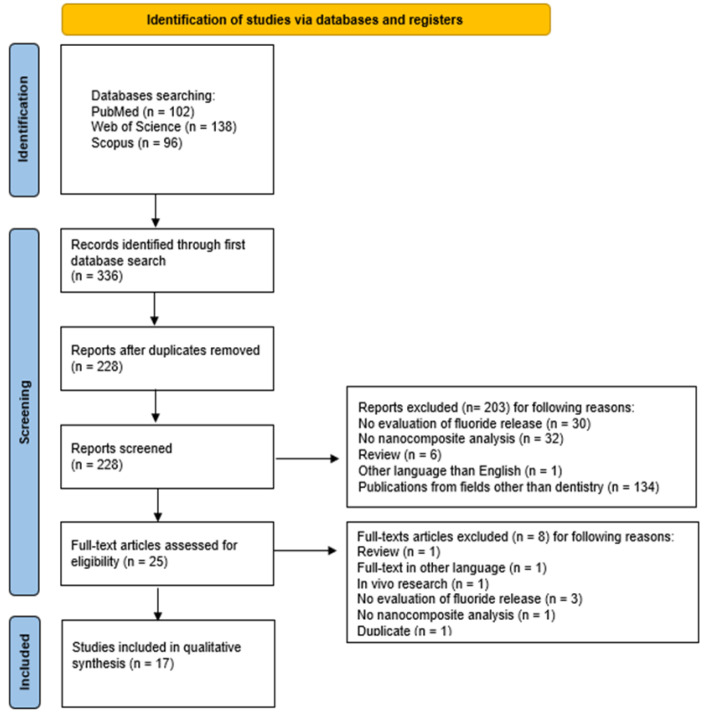
The PRISMA 2020 flow diagram.

The selection process for articles included in the systematic review was carefully outlined following the PRISMA flow diagram [47] (Figure 2). The systematic review was registered on the Open Science Framework under the following link: https://osf.io/mj4by (accessed on 12 March 2025).

### 2.3. Eligibility Criteria

The researchers decided to include only the articles that fulfilled the following criteria [47,48,49,50,51]:

- Nanocomposite dental materials;
- Fluoride release evaluation;
- In vitro studies;
- Studies in English;
- Full-text articles.

The reviewers established the following exclusion criteria [47,48,49,50,51]:

- Other than nanocomposite dental materials;
- Evaluation of other physical, chemical, or mechanical properties without evaluation of fluoride release;
- Non-English papers;
- Systematic review articles;
- Review articles;
- No full-text accessible;
- Duplicated publications.

No restrictions were applied with regard to the year of publication.

### 2.4. Information Sources, Search Strategy, and Study Selection

In February 2025, a systematic search was performed in the PubMed, Scopus, and Web of Science (WoS) databases to identify articles that met the specified inclusion criteria. To focus on factors affecting fluoride release from nanocomposites dental materials, the search was limited to titles, keywords, and abstracts using the following combination of key words: fluoride AND release AND (nanocomposite OR “nanostructured composite” OR “nanomaterial-based composite” OR “hybrid nanocomposite” OR “nanoengineered composite” OR “nanophased composite” OR “nanofilled composite”). All searches followed the predefined eligibility criteria, and only full-text articles available for access were included.

### 2.5. Data Collection Process and Data Items

Five independent reviewers (A.M.-W., A.K., J.K., S.K., and M.M.) carefully identified articles that fulfilled the inclusion criteria. The collected data encompassed the first author’s name, year of publication, study design, article title, nanocomposite dental restoration, and its fluoride release. All pertinent details were systematically documented in a standardized Excel file.

### 2.6. Risk of Bias and Quality Assessment

During the initial phase of study selection, each reviewer independently examined the titles and abstracts to reduce potential bias. The level of agreement among reviewers was assessed using Cohen’s kappa test. Any differences in opinion regarding the inclusion or exclusion of an article were resolved through discussion among the authors [52].

### 2.7. Quality Assessment

Two reviewers (J.M., M.D.) independently evaluated the quality of the selected studies. The evaluation criteria focused on aspects such as study design, execution, and analysis, including the following details: randomization, sample size calculation, control group, detailed description of material preparation in cases of studies when authors were not using commercial material, providing information about ion release measurement method, utilization of TISAB—Total Ionic Strength Adjustment Buffer, and sample storage method (environment pH/temperature/kind of solution—3 points for providing all 3 parameters, for two—2 points, for one—1 point). The studies were scored on a scale of 0 to 9 points, with a higher score indicating better study quality. The risk of bias was assessed as follows: 0–3 points indicated a high risk, 4–6 points indicated a moderate risk, and 7–9 points indicated a low risk. In cases where commercial composite was used so the authors were not able to provide material preparation description, the scale was 0–2 points high risk, 3–5 points moderate risk, and 6–8 points low risk of bias. Any discrepancies in scoring were resolved through discussion until a consensus was reached.

## 3. Results

### 3.1. Study Selection

The study selection process according to the PRISMA checklist is presented in Figure 2. The initial electronic database search across PubMed, Scopus, and WoS yielded 336 potentially relevant articles. After removing 108 duplicates, 228 unique records underwent initial screening. The screening of titles and abstracts resulted in the exclusion of 203 articles that did not meet the inclusion criteria: in 30 studies, no fluoride release evaluation was performed; 32 studies used materials other than nanocomposites; 6 studies were reviews; 1 study was written in a non-English language; and 134 studies were from other fields than dentistry. Thus, 25 publications were left for full-text review, during which 8 articles were excluded for the following reasons: 1 more study was a review; the full-text of 1 article was unavailable in English language; 1 article presented an in vivo study; in 3 studies, the fluoride release was not measured; in 1 study, the composite used was not present in the nanometric form; and 1 other study was a duplicate. Therefore, a total of 17 articles were included in this review.

### 3.2. General Characteristics of the Included Studies

Seventeen studies were included in this review. The general characteristics of the studies are presented in Table 1. The primary objective of most studies was to assess the fluoride ion release profile from nanomaterial-based composites. The main purpose of 15 research works was to develop novel composite materials to release fluoride [15,16,19,25,53,54,55,56,57,58,59,60,61,62,63]; the other two researchers analyzed commercially available materials only [61,62]. Most authors decided to compare nanocomposites with various fluoride-releasing materials; as a comparative control, they used materials such as conventional glass ionomer GIC in three cases [19,54,64] and resin modified glass ionomer RMGI [60,64]. Of these, only the polymer–kaolinite nanocomposite resins produced by Wang L-Y. et al. [54] showed higher fluorine release than GIC. Commercial composites were selected as a control group by eight authors, five of whom chose Heliomolar (IvoclarVivadent) [15,56,57,59,60,62,64]. Another three investigators presented fluoride release results in comparison to those reported in the literature for conventional materials [16,25,58], two of which obtained matching outcomes [16,25]. However, results obtained by Khan et al. [58] showed smaller and slower F^−^ ion release than conventional GI materials.

A total of 11 researchers, based on the assessment of fluoride release, reached conclusions regarding anticariogenic features [25,53,62,63] such as antibacterial properties [62,64], demineralization inhibition [15,56], and enamel remineralization promotion [58,60,61]. Interestingly, most authors concluded that the tested materials have anti-caries potential [15,16,49,57,60,61,62,63,64], while two others concluded that the presented properties are insufficient to prevent biofilm colonization or inhibit secondary caries [64,65]. Liu J. et al. investigated research to assess the biocompatibility of nano-CaF_2_ composites in the context of the restoration of root cavities in patients with periodontitis [55]. The fluoride ion release from their nano-CaF_2_ composite showed potential for promoting periodontal regeneration. The aim of the study by Khan A. S. et al. [58] investigated the nanocomposite as root canal filling material. The fluoride release values were significantly lower than the required values for oral health. The study by Leite et al. [63] was the only investigation that specifically used nanocomposite solutions to assess its effectiveness in preventing dental caries around orthodontic brackets bonded to bovine enamel. Komalsingsakul A. et al. [64] studied the effect of brushing on fluoride release from restorative materials. Two authors used nanocomposites for the purpose of a pit and fissure sealing [59,62]. Li K-Y. et al. analyzed fluoridated montmorillonite (FMMT) nanocomposite as a fissure sealant [59]. They came to the conclusion that the resin containing FMMT has excellent fluoride ion release and recharging properties. Following Fei X. et al. [62], despite the presence of CaF_2_ in the sealant significantly increasing the release of fluoride ions, the additional presence of DMAHDM slightly reduces the intensity of this phenomenon.

Furthermore, a group of nine authors assessed additional mechanical properties of prepared materials with nanoparticles [15,16,25,49,56,57,59,60,62]. However, a comparison of these parameters was not the subject of this review (see Supplementary Table S1.)

### 3.3. Main Study Outcomes

The objective of this review was to evaluate factors influencing fluoride release. The main result of the study is the finding that, despite differences in composition and material parameters, nanocomposites are capable of releasing fluoride ions. The detailed characterization of the studies is presented in Table 1. In the included studies, nanocomposites that release fluoride were used; however, the composition of the materials examined differed throughout the articles analyzed.

#### 3.3.1. Sample Size/Volume and Material Composition

The sample dimensions across the reviewed studies show considerable heterogeneity, ranging from very small discs (1 mm × 0.2 mm) [53] to larger rectangular blocks (15 mm × 15 mm × 1 mm) [58]. Several studies—including those by Mitwalli et al., Xu et al., Dai et al., Liu et al., and Fei et al.—used similarly shaped bar or cuboidal specimens (e.g., 2 mm × 2 mm × 12 mm) [15,16,25,55,56,57,60,62], while others employed disc-shaped samples of various diameters and thicknesses [19,54,59,61,64]. Notably, some studies, such as those focused on orthodontic bracket bonding, did not provide explicit sample dimensions [63,65]. The variation in sample geometry and surface area is a known factor influencing the rate and extent of ion release. Nevertheless, 8 out of the 17 studies followed comparable protocols for fluoride release testing, which enhances the reliability of cross-study comparisons [15,16,25,55,56,57,58,60,62].

All the studies involved in the review concerned fluoride release sources—whether CaF_2_ nanoparticles (nCaF_2_,) synthesized via spray-drying process in eight reports [15,16,25,56,57,58,60,62], or fluoridated hydroxyapatites/nano fluorapatites (FHA, nFA) in three studies [53,58,61]. The vast majority of the experimental materials were BisGMA and TEGDMA resin-based composites [15,16,25,53,54,55,56,57,60,62]. The analyzed works can be divided into two main groups: the first concerns materials with a constant composition of the resin matrix with a variable content of fluorine compounds [15,16,55,58,61]; the second group contains materials with a constant content of fluorinated compounds and a variable resin matrix composition [15,54,57,60,62]. As Meng L. et al. [61] prove, the release of fluoride is proportional to the initial F ion percentage in the material tested—the higher the levels of fluorine doping in adhesives, the higher the level of ion release [61]. Fei X. et. al. [62] obtained a lower cumulative amount for 5%DMAHDM + 20%nCaF than for a material with 0% DMAHDM and the same nCaF_2_ content. Leite K. L. F. et al. [63] prepared exceptionally mesoporous silica-based nanocomposites with the addition of titanium tetrafluoride and sodium fluoride, with or without calcium.

#### 3.3.2. Storage Conditions

Publications varied with each other in terms of the assessed storage conditions. The most common storage liquid was NaCl solution in eight cases [15,16,25,55,56,57,58,60,62], followed by deionized water (DW) in three publications [54,58,65] and artificial saliva (AS) mentioned two times [19,58]. Eight of the included studies used the same protocol where the specimens were stored in 50 mL of NaCl to obtain the sample volume-to-solution ratio of 2.9 mm^3^/mL [15,16,19,25,55,56,57,58,60]. A study led by Khan A. S. et al. [58] showed insignificant differences for DW and AS. Other fluids of choice were simulated body fluid (SBF) proposed by Taheri M. M. et al. [53] and Sayyedan F. S. et al. [19], and distilled water in a study published by Li K-Y. et al. [59] and Meng L. et al. [61]. Certain studies analyzed the problem in relation to biofilm formation. Due to that, Melo M. A. S. et al. [65] and Leite K. L. F. et al. [53] incubated the specimens with specific biofilm in Brain Heart Infusion medium (BHI) containing sucrose. The samples were stored in an environment with a pH of 4 to 7.4. The experiment by Xu H. H. K. et al. [25] was the only one conducted at three different pH values (pH = 4, 5.5, 7). The presented results showed that the cumulative F release increased with decreasing pH and increasing nano-CaF_2_ content. Not all authors took into account the temperature of sample storage; for the nine who did take this parameter into account, it was 37 °C [19,25,53,55,58,61,62,63,64].

#### 3.3.3. Measurement Methods

The most common method of fluoride measurement was an ion-selective electrode in combination with a reference electrode [15,16,19,25,54,55,56,57,58,60,62,63,64,65]. Ion chromatography was used by Taheri M. M. et al. [53] and Li K-Y. et al. [59]; the authors listed did not provide any numerical data. A review of full-text articles revealed heterogeneity across articles with respect to study duration, varying from 24 h [64] to 180 days [58]. The fact that fluorinated materials may have recharging ability has not escaped the attention of some researchers. Mitwalli et al. [60] determined that CaF_2_ nanocomposites with a relevant resin matrix composition have the potential to recharge and release F-ions and Ca- ions over a long period of time. The highest number of primary and secondary ions was released by the material containing pyromellitic glycerol dimethacrylate (PMGDM) and ethoxylated bisphenol A dimethacrylate (EBPADMA). The repeatable pattern of fluoride ion release was a high burst on the first day, followed by a slow decline in values on the following days. Sayyedan F. S. et al. [19] reported that their nanocomposite showed highest fluoride release on day 1, decreasing over time, similar to the GIC. Similarly, the release of F^−^ shown by Xu H. H. K. et al. [25] decreased with time (see Table 1).

#### 3.3.4. Fluoride Release Results

Despite the use of similar measurement methods, the results across the reviewed studies were reported in different units of measurement. The SI unit for expressing the concentration of a substance is moles per liter (mol/L), and the most frequently used unit for fluoride ion concentration was millimoles per liter (mmol/L), as reported by Mitwalli H. et al. [15,57,60], Xu H. H. K. et al. [25], and Fei X. et al. [62]. These authors typically reported cumulative fluoride concentrations measured at the end of the experimental period, which ranged from 70 days to 6 months. Due to the variation in measurement duration and differences in the chemical composition of the tested materials, a direct comparison of the reported values is not feasible. To allow for standardization, fluoride concentrations expressed in mmol/L were converted to mg/L using the molar mass of fluoride (19 g/mol). As per standard unit conversions, the units mg/L, μg/mL, and ppm are numerically equivalent [19,58,63,65]. Although these equivalent units were used in multiple studies, such as those by Khan A. S. et al. [58], Leite K. L. F. et al. [63], Sayyedan F. S. et al. [19], and Komalsingsakul A. et al. [65], differences in study design and testing protocols still preclude a direct comparison of results. Moreover, five studies did not provide any numerical values of fluoride release but instead presented their findings in qualitative or descriptive form: Taheri M. M. et al. [53], Wang L-Y. et al. [54], Liu J. et al. [55], Li K-Y. et al. [59] and Meng L. et al. [61].

#### 3.3.5. Estimated Daily Fluoride Release (Converted to μg/cm^2^/day)

A comparative analysis of fluoride release rates (in μg/cm^2^/day) showed substantial variability depending on material type and formulation. To allow cross-study comparison, all available data were recalculated into standardized daily release units. Values originally reported in mmol/L were converted to mg/L using the molar mass of fluoride (19 g/mol), then to total μg based on storage volume (typically 50 mL), and finally normalized to surface area (commonly 2.4 cm^2^) and time. Results expressed in ppm, mg/L, or μg/mL were treated equivalently. For cumulative values (e.g., μg/cm^2^ over several days), the average daily release was obtained by dividing by the study duration.

The highest fluoride release was observed for glass ionomer-based systems. In particular, the Fuji II GIC modified with forsterite nanoparticles showed extreme values, reaching 416,667 μg/cm^2^/day, while its nanocomposite counterpart released 372,024 μg/cm^2^/day [19]. Resin-modified glass ionomers (RMGIs), such as Vitremer, also demonstrated high release levels, with an initial release of up to 16,342 μg/cm^2^/day [60]. Among resin-based nanocomposites, materials containing nano-CaF_2_ combined with functional monomers such as DMAHDM and MPC exhibited significantly enhanced fluoride release. Mitwalli et al. reported release rates of 2261.9 μg/cm^2^/day for nCaF_2_ + MPC and 1413.7 μg/cm^2^/day for nCaF_2_ + DMAHDM + MPC [15]. Similarly, Fei et al. found that composites with 0% DMAHDM + 20% nCaF_2_ released 1364.0 μg/cm^2^/day, while those with 5% DMAHDM released 1002.9 μg/cm^2^/day [62]. The beneficial effects of DMAHDM were also reflected in other formulations, such as BT + nCaF_2_ + DMAHDM, which reached 5032.7 μg/cm^2^/day [57]. In contrast, commercial composites such as Heliomolar consistently showed the lowest fluoride release, typically below 0.1 μg/cm^2^/day [16,56]. Polyurethane-based composites containing nano-fluorapatite (PU/nFA) released approximately 0.30 μg/cm^2^/day [58]. Furthermore, a study by Melo et al., which measured fluoride accumulation in biofilm rather than direct elution, yielded a recalculated estimate of only 0.0362 μg/cm^2^/day [65]. It is important to note that some studies included in this review did not report fluoride release in numerical form. For example, Wang et al. [54], Liu et al. [55], and Meng et al. [61] presented their findings qualitatively or as relative comparisons to control materials. In these studies, fluoride release was described as “sustained”, “detectable”, or “higher than control”, often illustrated via figures or SEM/EDS analyses, without specifying absolute fluoride concentrations in μg/cm^2^/day or mmol/L.

### 3.4. Quality Assessment

**Figure 3:**
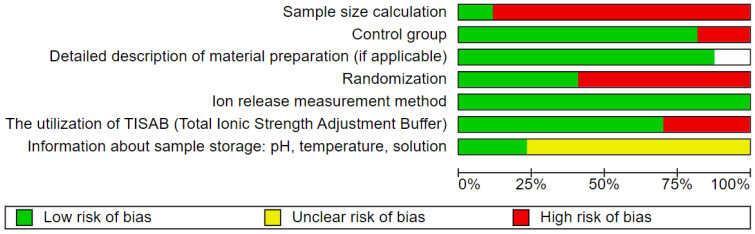
Quality assessment.

Among the articles included in the review, five [25,55,61,62,63] were rated as high-quality, achieving a score between 7 and 9 points out of 9. Twelve studies [15,16,19,53,54,56,57,58,59,61,64,65] were identified as having a moderate risk of bias, scoring between 4 and 6 points. Furthermore, none of the studies was categorized as low quality. A summary of the conducted quality assessment is presented in Figure 3.

## 4. Discussion

The principal objective of this systematic review was to provide a complete analysis of the factors affecting fluoride release from nanocomposite restorative materials, with particular focus on material composition [15,16,54,55,56,57,60,61,62], nanofiller properties [16,53,56,58,61], clinical conditions [63,65], and pH dynamics [16]. Most materials demonstrate a high initial fluoride release followed by a gradual decrease until reaching a stable lower release over time [15,16,19,25,55,56]. This pattern was observed across multiple studies regardless of the fluoride source or matrix composition. Fluoride-releasing components have variable effects on mechanical properties, with outcomes depending on specific nanoparticle type and concentration [15,53,59,62]. Higher concentrations of fluoride-containing nanoparticles, particularly 20% nCaF_2_, consistently increased fluoride release across multiple studies [16,55,56,57,58], reaching values of 1364.0 μg/cm^2^/day (Fei et al. [62]), 1413.7 μg/cm^2^/day (Mitwalli et al. [15]), and greater than 6500 μg/cm^2^/day in BT-based composites [57]. GIC-based materials such as Fuji II exhibited even more pronounced release, up to 416,667 μg/cm^2^/day [19], while commercial composites like Heliomolar remained consistently low, typically below 0.1 μg/cm^2^/day [16,56]. Some formulations exhibit the valuable property of fluoride rechargeability, allowing prolonged release after exposure to external fluoride sources [59,60]. Notably, the environmental pH appeared as a critical factor influencing release rates, with acidic conditions significantly increasing fluoride release [16]. The reviewed studies demonstrate that fluoride-releasing nanocomposites hold considerable potential for caries prevention, with the majority of researchers (11 out of 17) [15,16,25,53,54,57,59,60,61,62,64] concluding that their tested materials had sufficient anticariogenic properties to inhibit demineralization [15,65] promote remineralization [56,59,60,64], or exhibit antibacterial effects [62,64,65,66,67].

Material composition significantly influences fluoride release from dental nanocomposites. Studies included either calcium fluoride nanoparticles (nCaF_2_) [15,16,25,55,56,57,58,60,62] or fluoridated hydroxyapatites (FHA, nFA) [53,58,61]. Most experimental materials utilized Bis-GMA and TEGDMA as the resin matrix base [15,16,25,53,55,56,57,58,60,62], though some studies explored alternative matrices [19,54,58,59,60,62]. Multiple investigations confirmed that higher fluoride content consistently produced greater release rates [16,55,56,61]. Matrix composition proved fundamental, with BT-based resins presenting superior release compared to BTM-based formulations [57], while PMGDM/EBPADMA matrices demonstrated better recharging properties [60]. Polymer–kaolinite matrices showed promising results in specific formulations [54]. Functional additives impacted release patterns, with DMAHDM slightly reducing fluoride release [62] and MPC enhancing it [15]. The method of synthesis significantly affected results, with spray-dried particles outperforming those made by co-precipitation [16,25,56], confirming that nano-sized particles yielded better release characteristics than larger ones. In comparisons with conventional materials [19,25,54,64], only polymer–kaolinite nanocomposites exceeded GIC release rates [54]. Novel formulations included fluoridated montmorillonite with excellent recharging properties [59] and mesoporous silica nanocomposites with superior anticariogenic effects despite lower fluoride release [63], while Sayyedan et al. [19] found that adding forsterite nanoparticles to GIC slightly reduced fluoride release but enhanced bioactivity.

The storage conditions of the samples tested in the seventeen studies evaluated varied. Up to six different types of media were used to store the tested materials. In eight studies [15,16,25,55,56,57,58,60,62], the composite was incubated in NaCl solution. These studies were based on a standardized protocol in a previous study by O’Donnell et al. [68], where the tested specimens presented the same size and volume and were stored in the same volume of NaCl, maintaining the same sample volume-to-solution ratio. Thanks to this protocol, we obtain a homogeneous research model, by which the comparison of results becomes more reliable and burdened with a lower risk of bias. Despite the NaCl solution, three studies [54,58,64] stored the samples in deionized water, two studies [19,58] in artificial saliva, and two studies [59,61] in distilled water. In the case of [19,53], simulated body fluid (SBF) was used, and, in two papers where the issue of biofilm formation was additionally analyzed [63,65], the material was incubated in Brain Heart Infusion (BHI) with sucrose. Only one study compared the release of F-ions depending on the type of fluid in which the tested material was incubated. Khan A. S. et al. [58] showed insignificant differences in the release of F-ions from nanocomposites for deionized water (DW) and artificial saliva (AS). In the study by Xu H. H. K. et al. [16], NaCl solutions with different pH values (4; 5.5; 7) were prepared, which allowed them to show that the pH of the environment has an influence on the release of F ions from nanocomposites. It was significantly higher at pH 4 than at pH 5.5 or 7.

Of all the studies analyzed in the systematic review above, nine studies [19,25,53,54,58,61,62,63,64] considered the storage temperature of the tested materials. This parameter was not evaluated in eight studies [15,16,54,56,57,59,60,65]. In all studies where temperature was considered, it was 37 degrees Celsius. In this context, it should be noted that the nine studies mentioned above created conditions similar to the physiological conditions of the human oral cavity. Another parameter used to assess the storage environment of the samples was the pH of the solutions, which ranged from 4 to 7.4 and was determined in ten studies [15,16,25,55,56,57,58,59,60,62]. Only one study by Xu H. H. K. et al. [16] investigated the effect of changing the pH of the environment on the release of F-ions. For this purpose, the release of F-ions was evaluated at pH 4, 5.5, and 7. It was shown that the cumulative release of F-ions increases with decreasing pH. This provides evidence that the anticariogenic effect of nanocomposites increases with the acidity of the environment.

The review of studies evaluating the ability of nanocomposites to release fluoride ions showed a large heterogeneity in terms of the environmental conditions in which the materials were tested. One of the major limitations is that six different media were used in seventeen studies. The environment in which fluoride ions are released can have a major impact on the amount and dynamics of their release over time. Another important parameter not considered in eight studies was the temperature of the environment in which the materials were placed. Conditions similar to those found in the oral cavity would best simulate the environment in which fluoride ions are actually released from composites. In order to assess the potential usefulness and efficacy of the experimental nanocomposites, future studies should be conducted under uniform environmental conditions. The value of future studies in the context of assessing the anticariogenic effect of nanocomposite materials could be increased by taking into account the influence of changes in environmental pH on the release of F-ions. In the above review, only one in seventeen studies assessed the change in this parameter.

## 5. Conclusions

The systematic review of seventeen studies evaluating factors influencing fluoride ion release from dental nanocomposites demonstrated that several newly developed formulations released higher levels of fluoride compared to commercial composites, which consistently showed minimal release (<0.1 μg/cm^2^/day). However, none of the nanocomposite materials matched the fluoride release capacity of conventional glass ionomer cements (GICs), with values reaching up to 416,667 μg/cm^2^/day in modified GICs. The majority of tested nanocomposites exhibited anticaries potential, supported by in vitro findings of reduced demineralization, remineralization promotion, and, in some cases, antibacterial effects. Importantly, these effects were achieved without compromising mechanical integrity. Additionally, certain experimental materials demonstrated fluoride rechargeability and potential benefits for periodontal regeneration and white spot lesion prevention around orthodontic brackets. A key limitation of this review is the heterogeneity of study protocols, including differences in storage media, sample dimensions, duration, and units of measurement, which restricts a direct comparison of outcomes. The standardization of testing conditions and analytical methods is essential for future studies to enable reliable cross-study comparisons. Furthermore, since all included studies were conducted under laboratory conditions, there remains a critical need for clinical or in situ investigations to validate their real-world applicability.

## Figures and Tables

**Table 1:** Detailed characteristics of included studies. N/A-no information.

Authors	Sample Size/Volume	Material Composition	Measurement Method	Storage Conditions	Fluoride Release Results	Fluoride Release Rate (μg/cm^2^/day)
Taheri [53]	1 mm × 0.2 mm	Bis-GMA/TEGDMA + HA + NaF + I-819	Ion chromatography	- Simulated body fluid (SBF), - Composite surface area-to-volume: 10 mm^2^/mL - 37 °C - 21 days	0.97 ppm (μg/mL) at 21 days for 0.2 wt% FHA	138.6
Melo [65]	N/A	Orthocem (Dentscare Ltd.a., Joinville, SC, Brazil)	Fluoride-sensitive electrode	- *S. mutans* biofilm	1.48 (1.24) μg/g wet Biofilm Incubation time 5 days	0.0362 (estimated; based on 1.48 μg/g wet biofilm, assuming 0.012 g biofilm mass and 0.09806 cm^2^ bracket contact area over 5 days)
Wang [54]	6 mm diameter × 2 mm thickness	Kaolinite + diamine/acrylamide/acetate + Bis-GMA/TEGDMA (NaF only in diamine)	Ion-selective electrode	- 3 mL of deionized water - 56 days	No numeric data	No numeric data
Mitwalli [15]	2 mm × 2 mm × 12 mm	BT + CaF_2_ (+DMAHDM/+MPC/+DMAHDM + MPC) + glass	Ion-selective electrode	- 50 mL NaCl solution - pH 7 - 70 days	nCaF_2_ + MPC = 0.40 ± 0.02 mmol/L nCaF_2_ + DMAHDM + MPC = 0.25 ± 0.03 mmol/L nCaF_2_ + DMAHDM = 0.20 ± 0.03 mmol/L nCaF_2_ = 0.04 ± 0.01 mmol/L	nCaF_2_ + MPC: 2261.90 nCaF_2_ + DMAHDM + MPC: 1413.69 nCaF_2_ + DMAHDM: 1130.95 nCaF_2_: 226.19
Dai [56]	2 mm × 2 mm × 12 mm	Heliomolar (Ivoclar); Exp.: Bis-GMA/TEGDMA + CaF_2_ + glass + silane	Ion-selective electrode	- 50 mL NaCl solution - specimen volume of solution: 2.9 mm^3^/mL - pH = 7 - 84 days	20% CaF_2_cp composite = 183.7 ± 5.8 g/cm^2^ 20% nCaF_2_cpsd composite = 103.6 ± 9.4 g/cm^2^ Heliomolar = 1.6 ± 0.1 g/cm^2^	20% CaF_2_cp composite: 2.19 20% nCaF_2_cpsd composite: 1.23 Heliomolar: 0.019
Xu [16]	2 mm × 2 mm × 12 mm	Heliomolar (Ivoclar); Exp.: CaF_2_ + glass + Bis-GMA/TEGDMA + silane + additives	Ion-selective electrode	- 50 mL NaCl solutions - specimen volume of solution: 2.9 mm^3^/mL - pH = 4, 5.5, or 7, - 84 days	Nanocomposite30CaF_2_ = 327 ± 8 μg/cm^2^ Nanocomposite20CaF_2_ = 252 ± 8 μg/cm^2^ Nanocomposite10CaF_2_ = 47 ± 2 μg/cm^2^ Heliomolar = 4.7 ± 0.1 μg/cm^2^	Nanocomposite 30% CaF_2_: 3.89 Nanocomposite 20% CaF_2_: 3.00 Nanocomposite 10% CaF_2_: 0.56 Heliomolar: 0.056
Mitwalli [57]	2 mm × 2 mm × 12 mm	Heliomolar (Ivoclar); BT/BTM + CaF_2_ ± DMAHDM + glass	Ion-selective electrode	- 50 mL NaCl solution - specimen volume of solution: 3.0 mm^3^/mL - pH 4 - 70 days	BT + nCaF_2_ = 1.15 ± 0.01 mmol/L BT + nCaF_2_ + DMAHDM = 0.89 ± 0.01 mmol/L BTM + nCaF_2_ = 0.44 ± 0.01 mmol/L BTM + nCaF_2_ + DMAHDM = 0.22 ± 0.03 mmol/L Heliomolar = 0.02 ± 0.0008 mmol/L	BT + nCaF_2_: 6503 BT + nCaF_2_ + DMAHDM: 5033 BTM + nCaF_2_: 2488 BTM + nCaF_2_ + DMAHDM: 1244 Heliomolar: 113.10
Komalsingsakul [64]	5 mm diameter × 2 mm thickness	Filtek Z350XT (3M ESPE, St. Paul, MN, USA)	Ion-selective electrode	- 1 mL of deionized water - 37 °C - 24 h	Without brushing = 0.0032(0.0015) ppm With brushing = 0.0040(0.0026) ppm	Z350 XT (without brushing): 66.67 Z350 XT (with brushing): 83.33
Xu [25]	2 mm × 2 mm × 12 mm	1. Experimental composite: Bis-GMA, TEGDMA 2. Commercial control (TPH, Caulk/Dentsply, Milford, DE, USA)	Ion-selective electrode	- 50 mLNaCl solution - specimen volume of solution: 2.9 mm^3^/mL - pH = 7.4; 3 - 7 °C, 70 days	The cumulative F release (0.15 ± 0.03) mmol/L at 1 day = (0.15 ± 0.03) 1 week = (0.68 ± 0.11), 10 weeks = (2.34 ± 0.26)	Average 21.84
Liu [55]	2 mm × 2 mm × 12 mm	Heliomolar (Ivoclar); Exp.: BT + glass ± 10–20% nCaF_2_	Ion-selective electrode	- 50 mL NaCl solution - specimen volume of solution: 2.9 mm^3^/mL - pH = 7 - 37 °C - 56 days	No numeric data	No numeric data
Khan [58]	15 mm × 15 mm × 1 mm	PU composites + 10–20% nFA (nano-fluorapatite)	Orion Ionplus fluoride electrode	- 12 mL of artificial saliva (AS) and 12 mL of deionized water (DW) - pH = 6.8 - 37 °C, - 180 days	1 day = 0.0003 mg/L 7 days = 0.00094 mg/L The total release at 6 months = 0.0026 mg/L	Average 0.30
Li [59]	6 mm diameter × 2 mm thickness	ClinproTM (3M, Maplewood, MN, USA) Fluorinated montmorillonite (FMMT)	Ion chromatography	- 5 mL of distilled water - Recharging: 5 mL of 0.2% aqueous NaF at pH 7 - 1 min	No numeric data	No numeric data
Mitwalli [60]	2 mm × 2 mm × 12 mm	Vitremer (RMGI, 3M) Ketac Nano (RMGI, 3M) Heliomolar (fluoride composite, Ivoclar) Bis-GMA/TEGDMA Bis-GMA/TEGDMA + Bis-MEP PMGDM/EBPADMA	Selective electrode	- 50 mL NaCl buffered with lactic acid to pH 4 duration time: 70 days - specimen volume of solution: 2.9 mm^3^/mL - Recharging: 5 mL solution contained F ion concentration of 5000 ppm, pH = 7, duration time: 1 min	Initial (mmol/L): Vitremer 2.89, PE-nCaF_2_ 1.28, BT-nCaF_2_ 1.15, BTM-nCaF_2_ 0.44, Heliomolar 0 After 6 cycles: Vitremer 1.29, PE-nCaF_2_ 0.63, BT-nCaF_2_ 0.28, BTM-nCaF_2_ 0.26, Heliomolar 0.02 No recharge: Vitremer 1.96, PE-nCaF_2_ 1.19, BT-nCaF_2_ 0.79, BTM-nCaF_2_ 0.50 At 98 days: Helioseal F 0.0098, 0% DMAHDM 0.3377, 5% DMAHDM 0.2483	Vitremer: 16,342 PE-nCaF_2_: 7238 BT-nCaF_2_: 6503 BTM-nCaF_2_: 2488 Heliomolar: 0
Meng [58]	10 mm diameter × 1 mm hight	Adhesive (MPA-epoxy) ± FHA nanorods (2–10% F)	IC (ICS-5000+, Thermo Fisher Scientific, USA) measurer	- 2 mL of DI water, placed in a 37 °C shaker - duration time: 30 days	No numeric data	No numeric data
Fei [62]	2 mm × 2 mm × 12 mm	Helioseal F (Ivoclar); Exp.: 20% nCaF_2_ ± 5% DMAHDM	Combination of a fluoride ion-selective electrode and a reference electrode	- 50 mL of NaCl solution (133 mmol/L) buffered with 50 mmol/L HEPES (pH = 7.4; 37 °C) - specimen volume of solution: 2.9 mm^3^/mL - duration time: 98 days	Measured after 98. Day (cumulative amount): Helioseal F 0.0098 ± 0.0019 mmol/L 0% DMAHDM + 20%nCaF_2_ 0.3377 ± 0.0325 mmol/L 5%DMAHDM + 20%nCaF 0.2483 ± 0.0054 mmol/L	Helioseal F: 39.58 0% DMAHDM + 20% nCaF_2_: 1364.01 5% DMAHDM + 20% nCaF_2_: 1002.91
Sayyeda [19]	4 mm diameter × 6 mm thickness	GIC (Fuji II) + forsterite (Mg_2_SiO_4_)	Fluoride ion-selective electrode	- 15 mL Artificial saliva - room temperature - 14 days - simulated body fluid (SBF) - 37 °C - 21 days	Specific values: - day 1: ~130 ppm for Fuji II GIC, ~120 ppm for nanocomposite - day 3: ~85 ppm for Fuji II GIC, ~80 ppm for nanocomposite - day 7: ~45 ppm for Fuji II GIC, ~35 ppm for nanocomposite - day 14: ~20 ppm for Fuji II GIC, ~15 ppm for nanocomposite	Fuji II GIC: 416,667 Nanocomposite: 372,024
Leite [63]	N/A	MSCaTiF_4_: MS + TiF_4_ + Ca MSTiF_4_: MS + TiF_4_ MSCaNaF: MS + NaF + Ca MSNaF: MS + NaF	Fluoride ion-selective electrode	- Artificial saliva - 37 °C - 2 h - Multispecies biofilm (*S. mutans*, *S. salivarius*, *S. sanguinis* and *L. casei*)	Specific TSF values (μg F⁻/mL): MSCaTiF_4_: 3.52 ± 1.68, MSTiF_4_: 1.39 ± 0.60, MSCaNaF: 3.25 ± 0.48, MSNaF: 1.16 ± 0.31, TiF_4_: 19.76 ± 9.88, NaF: 10.18 ± 4.34. After 24 h	TiF_4_: 411.67 MSCaTiF_4_: 73.33 MSCaNaF: 67.71 MSTiF_4_: 28.96 MSNaF: 24.17

## Supplementary Materials

The following supporting information can be downloaded at: https://www.mdpi.com/article/10.3390/nano15090651/s1. Table S1. General characteristics of studies.
