# Supplementary material for: Evaluation of Factors Influencing Fluoride Release from Dental Nanocomposite Materials: A Systematic Review
Source: Nanomaterials (Basel). 2025 Apr 25;15(9):651. doi: 10.3390/nano15090651 (PMC12073368; doi:10.3390/nano15090651)
Supplement: Supplementary file 1 [file nanomaterials-15-00651-s001.zip › nanomaterials-3565051-supplementary.pdf]

This table provides a summary of the 17 studies included in the systematic review titled: **"Evaluation of Factors Influencing Fluoride Release from Dental Nanocomposite Materials: A Systematic Review"**

Authors:

Alicja Morawska-Wilk[1], Julia Kensy[2], Sylwia Kiryk[1], Agnieszka Kotela[3], Jan Kiryk[4], Mateusz Michalak[3], Natalia Grychowska[5], Magdalena Fast[6], Jacek Matys[4*] and Maciej Dobrzyński[1]

1 Department of Pediatric Dentistry and Preclinical Dentistry, Wroclaw Medical University, Krakowska 26, 50-425 Wroclaw, Poland; alicja.morawska@umw.edu.pl (A.M-L.); s.roguzinska@gmail.com (S.K.); maciej.dobrzynski@umw.edu.pl (M.D.)

2 Faculty of Dentistry, Wroclaw Medical University, Krakowska 26, 50-425 Wroclaw, Poland; julia.kensy@student.umw.edu.pl (J.K.)

3 Medical Center of Innovation, Wroclaw Medical University, Krakowska 26, 50-425 Wroclaw, Poland; mateusz.michalak92@gmail.com (M.M.); kotela.agnieszka@gmail.com (A.K.)

4 Dental Surgery Department, Wroclaw Medical University, Krakowska 26, 50-425 Wroclaw, Poland; jan.kiryk@umw.edu.pl (J.K.); jacek.matys@umw.edu.pl (J.M.)

5 Department of Dental Prosthetics, Wroclaw Medical University, Krakowska 26, 50-425 Wroclaw, Poland; natalia.grychowska@umw.edu.pl (N.G.)

6 Department of Drug Form Technology, Wroclaw Medical University, Borowska 211 A, 50-556 Wroclaw, Poland; magdalena.fast@umw.edu.pl (M.F.)

* Correspondence: jacek.matys@umw.edu.pl

Table S1. General characteristic of included studies.

Study	Aim of the study	Material and Methods	Results	Conclusions
Taheri [50]	Evaluation of mechanical properties and fluoride ion release profile and pH changes in composites for dental applications with fluoridated hydroxyapatite (FHA) nanotubes.	FHA nanotubes were synthesized and incorporated into a composite matrix in various proportions to investigate the fluoride ion release and pH changes in simulated body fluid (SBF). The resin without FHA served as a control sample.	Reinforcing the composite with 0.2 wt% FHA improved its mechanical properties. Additionally, fluoride ion release lowered the pH in SBF.	Lowering the pH due to the release of fluoride ions may contribute to the prevention of caries.
Melo [61]	To investigate the fluoride concentration in *S. mutans* biofilm formed around orthodontic brackets bonded with a fluorinated composite containing nanofillers.	Samples were prepared from the following materials: natural ortho, orthodontic fill magic, vitremer and ortho cem and orthodontic brackets were bonded. The samples were placed in a *S. mutans* bacteria culture. Then the fluoride level in the bacterial plaque on the surface of each sample was tested.	Nanofilled composite released more fluoride than microfilled composite. However, the difference was not statistically significant.	Adding nanofillers to fluoride composite resin does not increase the inhibition of demineralization.
Wang [51]	Development of a composite resin for dental fillings with high fluoride release and loading properties.	Samples were prepared from the following materials: C(K-diamine), C(K-acrylamide), C(K-acetate), Fuji IX and Z-100. Fluoride levels were measured using an ion-selective electrode after storage in deionized water for 56 days.	All the produced polymer-kaolinite nanocomposite resins showed higher fluorine emission than Fuji IX resin.	The fluoride releasing and loading properties of C(K-acrylamide) were better than those of Fuji IX and Z-100.
Mitwalli[15]	To develop a new composite consisting of calcium fluoride (nCaF2), dimethylaminohexadecyl methacrylate (DMAHDM) and 2-methacryloyloxyethylphosphorylcholine (MPC) and to investigate its mechanical properties, ion release and effect on oral biofilm.	Samples of Heliomolar, nCaF2, nCaF2+DMAHDM, nCaF2+MPC and nCaF2+DMAHDM+MPC were placed in NaCl at pH 7 for 70 days. The amount of released fluorine was measured using an ion-selective electrode.	At 70 days, the composite nCaF2+MPC had the highest F release of (0.40 ± 0.02) mmol/L while nCaF2+DMAHDM+MPC released (0.25 ± 0.03) mmol/L, nCaF2+DMAHDM (0.20 ± 0.03) mmol/L, and nCaF2 (0.04 ± 0.01) mmol/L.	The release of F ions from the bioactive nanocomposite was achieved by incorporating nCaF2. It has the potential to protect tooth structures, inhibit demineralization and provide release of fluoride ions.

Dai [53]	Development of a new light-cured $nCaF_2$ nanocomposite with strong mechanical properties and high F release.	Samples were prepared from 20%$nCaF_2$cpsd composite, 20%CaF_2cp composite, and commercial control and stored for 84 days in NaCl solution at pH7. Fluoride release was measured with an ion-selective electrode.	The highest amount of fluorine was obtained from 20% $nCaF_2$cpsd composite and the lowest from commercial control.	The 20% $nCaF_2$cpsd composite showed 65-fold higher cumulative F ion release and 77-fold higher long-term F ion release rate than the commercial composite.
Xu [16]	To investigate the effects of nano-CaF_2 filler level and solution pH on fluoride release and mechanical properties of CaF_2 nanocomposites with glass reinforcement.	Samples containing 10%, 20% and 30% (mass%) of nano-CaF_2 were fabricated in the form of flowable paste and incubated. Commercial composite with nano-sized fillers (Heliomolar) and resin-modidfied Glassionomer (Vitremer) were employed as a control. Specimens were stored in NaCl solution at pH 4, pH 5.5 and pH 7 for 84 days. The fluoride concentration was measured with an ion-selective electrode.	The cumulative F release increased with decreasing pH and increasing nano-CaF_2 filler level. At 84 days and pH 4, fluoride release showed the highest value for Nanocomposite30CaF_2, Vitremer had the highest F release from all samples. All materials showed a high initial F release, followed by a lower, steady-state release. The F-release rate decreased with increasing time for 7-9 weeks. The F-release rate at pH 4 was higher than those at pH 5.5 and pH 7 for the first 4-6 weeks.	The nano-CaF_2 composites produced high F release at low filler levels. The fluoride release rate was equivalent to the ion release rate of a commercial resin-modified glass ionomer.
Mitwalli [54]	Development of a new composite material by incorporating dimethylaminohexadecyl methacrylate (DMAHDM) with remineralizing and antibacterial properties due to the content of F and Ca ions.	Samples made of Heliomolar, BT+$nCaF_2$, BTM+$nCaF_2$, BT+$nCaF_2$+DMAHDM and BTM+$nCaF_2$+DMAHDM composite were stored in NaCl solution at pH 4 for 70 days. Fluorine release was measured using an ion-selective electrode.<br><br>BT- bisphenol A glycidyl dimethacrylate (BisGMA) + triethylene glycol dimethacrylate (TEGDMA)<br>BTM- BisGMA+TEGDMA+Bis[2-(methacryloyloxy)ethyl] phosphate (Bis-MEP)	Composites with $nCaF_2$ alone had a greater release of F ions compared to composites with $nCaF_2$+DMAHDM. Composites with BT-based resin released more fluoride compared to composites with BTM-based resin.	Newly developed composites containing $nCaF_2$ and DMAHDM achieve high levels of F and Ca ion release, necessary for potential remineralization.
Komalsingsakul [62]	To investigate the surface roughness, fluoride release and *S. mutans* biofilm formation on dental restorative materials before and after brushing.	Samples were made from the following materials: Filtek Z350 XT, Beautifil II, GC Fuji II LC Capsule and GC Fuji IX GP Extra Capsule. After brushing or without, they were placed for 24 hours in deionized water at 37°C. Fluoride release was measured using an ion-selective electrode.	Fuji II LC and Fuji IX GP Extra released the highest amount of fluoride after brushing, followed by Beautifil II and Z350 XT.	The amount of fluoride released was material and time dependent, with the exception of Z350XT. Fluoride release from these materials was insufficient to prevent *S. mutans* colonization.
Xu [40]	To develop stress-bearing dental nanocomposites for tooth cavity restorations with caries-inhibiting capabilities.<br><br>Testing whether a resin composite containing CaF_2 nanoparticles will have a consistent release of high levels of fluorine.	Nanocomposite samples with 25% whiskers, 20% CaF_2, and 20% DCPA (total filler content of 65% by mass) were prepared. The specimens were then immersed in a NaCl solution at pH 7.4 and 37 °C for 70 days to measure the fluoride ion release concentrations.	The cumulative fluoride release was (0.15 ± 0.03) mmol/L on day 1 and gradually increased over the 10-week period. The initial fluoride release rate was 1.94 μg/(h·cm^2), which decreased over the course of the study.	The nanocomposite released fluoride ions at levels equal to or higher than those reported for traditional glass ionomer and resin-modified glass ionomer materials. Both the mechanical properties and fluoride ion release of the composite could be adjusted by modifying the amount of nanoparticles or reinforcing filler.

Liu [52]	To develop a biocompatible Nano-CaF_2 composite and assess its effects on osteogenic and cementogenic differentiation of periodontal ligament stem cells from human donors.	Specimens with 0%, 10%, 15%, and 20% Nano-CaF_2, along with Heliomolar (clinical control), were immersed in NaCl solution (pH 7, 37 °C). Fluoride ion concentrations released from the samples were measured over 56 days using a fluoride ion-selective electrode.	The 20% NanoCaF_2 composite exhibited the highest initial release of fluoride and calcium ions compared to all other groups. The fluoride release from the 20% NanoCaF_2 composite was 30 times greater than that of Heliomolar, initially decreasing from day 1 to day 14, before stabilizing.	The 20% CaF_2 composite seems to be ideal for its sustained ion release and strong load-bearing properties. The biocompatible Nano-CaF_2 composite shows potential for treating root cavities in periodontitis patients, promoting periodontal regeneration with calcium and fluoride ion release.
Khan [55]	Development of a nano-fluorapatite (nFA) root canal filling material that releases fluoride and minimizes tooth interfaces.	Samples including nFA powder, PU/nFA10, PU/nFA15 and PU/nFA20 were stored at 37 °C in centrifuge tubes containing artificial saliva (AS, pH 6.8) and deionized water (DW) for periodical time intervals, 180 days in total. The solutions were tested for fluoride content using Orion Ionplus Fluoride Electrode.	The nFA value showed an increasing trend of fluoride release over time for DW and AS, with no significant difference in the fluoride release pattern in both cases.<br>At 6 months the total release were 0.0026 mg/L.<br>The level of fluoride release in PU/nFA composites depended on the fluoride level in the composite, with no direct proportional relationship.	The observed fluoride release rate was smaller and slower than that reported for conventional materials such as glass ionomer cements. The fluoride release values were significantly lower than the required values for oral health. The tendency of fluoride release was almost the same in artificial saliva and deionized water.
Li [56]	Evidence that a fluorided montmorillonite (FMMT) nanocomposite fissure and pit sealant has sufficient mechanical properties and the ability to recharge and release fluoride ions over a long period of time.	Six samples of each of the resin with FMMT and the ClinproTM nanocomposite (control sample) were placed in deionised water to test the amount of fluoride that was initially released and re-released and the ability of the samples to recharge the F-ions. The mechanical properties of the materials were also tested: viscosity, hardness, toughness, tensile strength in diameter, flexural strength and wear resistance.	The resin containing FMMT had poorer mechanical properties than ClinproTM. The release and recharge of fluoride ions was higher in the FMMT resin than in ClinproTM.	The resin containing FMMT has excellent fluoride ion release and recharging properties. This makes it an excellent material for fissure and pit sealing.
Mitwalli [57]	To determine the composition of a rechargeable CaF_2 nanocomposite material and to investigate the possibility of re-releasing and recharging F and Ca while maintaining good mechanical parameters.	Three samples of CaF_2 nanocomposites with different resin matrix compositions and three samples as controls were subjected to flexural strength tests and their moduli of elasticity were evaluated. The release of Ca and F ions before and after recharging and the recharging ability of each sample were also evaluated.	Among all the samples, the CaF_2 nanocomposite with BisGMA and TEGDMA in the resin matrix exhibited the best mechanical properties. Among all the samples, Vitremer (RMGI) - the control group - had the highest primary and re-emission of F ions, and among the CaF_2 nanocomposites, the highest number of primary and secondary ions was released by the material containing pyromellitic glycerol dimethacrylate (PMGDM) and ethoxylated bisphenol A dimethacrylate (EBPADMA).	CaF_2 nanocomposites with a suitable resin matrix composition have the potential to recharge and release F and Ca ions over a long period of time. This demonstrates the long-term remineralisation potential of CaF_2 nanocomposites.
Meng [58]	To assess whether fluorine-substituted hydroxyapatites (FHA) nanotubes of different sizes and with different levels of fluoride doping in adhesives have an effect on the remineralisation of enamel and shear bond strength (SBS).	Three samples of the adhesive containing FHA nanotubes with fluorine contents of 2, 6 and 10% were used. In vitro studies measured the pH and Ca and F ion content of the supernatant formed after soaking the samples, as well as the morphology of the crystals precipitated on the samples. In vivo studies determined the chemical composition and morphology of enamel coated with the FHA nanotube adhesive.	In vitro: As the fluoride content in the nanotubes increased, the pH increased, the amount of Ca and F ions released and the amount of apatite crystals formed increased. In vivo: the more fluoride the adhesive contains, the better the mineralisation of the enamel.	Adhesive containing FHA nanotubes has the potential to remineralize enamel.

Fei [59]	To investigate whether a sealant containing CaF_2 and dimethylaminohexadecyl methacrylate (DMAHDM) enhances fluoride release and antimicrobial properties, and whether these compounds affect material mechanical properties.	The hardness (Vickers indenter with load) and fluoride release (measured using a combination of a fluoride ion selective electrode and a reference electrode) of three sealants were studied: 1. Helioseal F (control group)<br>2. 0% DMAHDM + 20% $nCaF_2$<br>3. 5% DMAHDM + 20% $nCaF_2$. The effect of sealants on the viability, metabolic activity, lactic acid production, colony formation and pH of *S. mutans* biofilm was also studied.	Adding CaF_2 and DMAHDM to the sealant increased the hardness of the material. The presence of CaF2 in the sealant significantly increases the release of fluoride ions, and the additional presence of DMAHDM slightly reduces the intensity of this phenomenon. A greater antibacterial effect and higher pH of the biofilm were also observed compared to Helioseal F (control group).	The addition of CaF2 and DMAHDM to the sealant gives the material anti-cariogenic and remineralising potential.
Sayyeda [19]	To investigate if adding nanoparticles to GIC improves its fluoride release and bioactivity.	Forsterite (Mg_2SiO_4) nanoparticles were created via sol-gel process and mixed 3% of these with GIC (Fuji II GC) creating a experimental nanocomposite. 3 samples of GIC and modified GIC material were placed in 15ml of artificial saliva. Fluoride release was measured at 1, 3, 7, 14 days using an ion-selective electrode. Measurements of bioactivity, calcium ion concentrations, pH level were additionally made.	Both materials released fluoride with the highest amount occurring in the first day, followed by a gradual decrease over the 14-day testing period. The nanocomposite (GIC with 3% forsterite nanoparticles) consistently showed lower fluoride release compared to the standard GIC (Fuji II).	The nanocomposite released less fluoride than the standard GIC, but showed increased bioactivity, exhibited better apatite formation on its surface, and faster silica gel layer formation was observed.
Leite [60]	To assess the effectiveness of nanocomposite solutions in preventing dental caries around orthodontic brackets	Randomized, controlled, single-blind in vitro study with fluoride-containing mesoporous silica nanocomposites (MS with TiF_4 or NaF) with or without calcium. Bovine enamel blocks with bonded orthodontic brackets were treated with test solutions (n=13 per group) and then exposed to a multi-species biofilm for 24 hours. Analyses included pH measurements, fluoride release, surface mineral loss, subsurface mineral volume and surface topography.	Nanocomposites released considerably less fluoride than conventional solutions. All nanocomposites demonstrated superior protection against demineralization compared to conventional fluorides. MSNaF maintained the highest culture medium pH, similar to $MSTiF_4$, while conventional fluoride controls showed significantly lower pH values.	A single application of mesoporous silica with fluoride offers superior protection for enamel around orthodontic brackets. The nanocomposites maintained higher pH levels, released fluoride more gradually, and provided superior protection against mineral loss while preserving enamel surface integrity.
